# Perceptions Regarding Tobacco Cessation Counselling among Dental Students and Graduates: A Cross-Sectional Study

**DOI:** 10.31557/APJCP.2019.20.9.2589

**Published:** 2019

**Authors:** Priyanka Kachwaha, Deepak Kumar Singhal, Nishtha Singh

**Affiliations:** 1 *Manipal College of Dental Sciences,*; 2 *Department of Public Health Dentistry, Manipal College of Dental Sciences, Manipal, Manipal Academy of Higher Education, Karnataka, India. *

**Keywords:** Tobacco cessation, counselling, dental professionals, role model, attitude

## Abstract

**Background:**

Tobacco usage is a global concern and it is essential to curb its usage and increase awareness among patients. Dental professionals’ contribution in tobacco cessation will definitely make a stark difference. Thus it is important to assess dental students’ and graduates’ knowledge, attitude and practice towards Tobacco cessation counselling (TCC).

**Materials and Methods:**

The descriptive, cross-sectional survey was carried out among 286 students of a dental college in South India. A well-structured, pretested, self-administered questionnaire consisting of 17 close-ended questions was employed to assess knowledge, attitude and practice towards TCC.

**Results:**

Almost all study participants agreed that tobacco cessation counselling is under the scope of dental practice and admitted that it is the responsibility of dental professionals to educate patients for the same. Majority of graduates responded that they have done tobacco cessation counselling whereas less than two-third of undergraduates have done the same and difference between two groups was statistically significant (p<0.001). A significantly higher number of graduates (79%) were aware of 5A’s and 5R’s protocol for TCC as compared to undergraduates (50%). But less than one-fifth of study population have actually applied this protocol for TCC in clinical practice.

**Conclusion:**

There is a need to modify the dental curriculum and motivate young graduates and students about TCC. This will develop a more professional competence and helps to encourage the dental professionals in developing a preventive mind set about tobacco use. Thus, it will help in enhancing the long-term rates of quitting tobacco among patients, which will prove beneficial in controlling tobacco related diseases in near future.

## Introduction

Tobacco use is single most preventable cause of morbidity and mortality globally (Rajasundaram et al., 2011; Salman et al., 2014). At present, tobacco usage is a global concern as the World Bank predicts over 450 million tobacco deaths in next 50 years. Tobacco cessation counselling by a health professional has been proven effective and it has been reported that appropriate assistance and professional advice by health professionals has led to a cessation rate of 10% -20% (Rajasundaram et al., 2011).

As oral screening and patient education forms an integral part of dental practice thus, dental clinic has been suggested as an ideal setup for tobacco cessation counselling (Rajasundaram et al., 2011). The prevention and control of tobacco use is an emerging issue of global significance and the important links between tobacco and oral health provide a unique opportunity for dentist to become involved in tobacco cessation activities. Various surveys in American and Canadian population showed that 58% of smokers visited their dentist at regular intervals (Yip et al., 2000; Rikard et al., 2003). Dental appointments can be used to educate such patients about harmful effects of tobacco and encourage them to quit the same. However, it has been found that dental professionals are not adequately trained with the tobacco cessation services and protocol as opposed to physicians and other health professionals (Rajasundaram et al., 2011).

Dental students, being the future dental professionals, examine the oral cavity and usually encounter medical emergencies in dental clinics (Singh et al., 2018). They often encounter the manifestations of tobacco on oral tissues ranging from stains on teeth, halitosis, premalignant lesions and cancerous lesions. Dental treatment that often necessitates multiple visits, provides a mechanism for initiation, reinforcement and support of tobacco cessation activities. Thus the aim of this study was to determine the knowledge, attitude and practice of tobacco cessation counselling among dental students and graduates.

## Materials and Methods

The descriptive, cross-sectional survey was carried out among 286 students, which included the graduates pursuing internship and undergraduates including third and final year students of a dental college in South India. This study assessed the knowledge, attitude and practice of tobacco cessation counselling among dental students and graduates.

The study population was selected as they come across patients using tobacco in the dental clinics and have been taught about tobacco cessation counselling as a part of their curriculum. The participation was voluntary and identity of participants was kept confidential. Informed consent was obtained from participants prior to filling up of questionnaire. Before conducting survey, permission was sought from Head of the Institution. Ethical clearance was also obtained from Institutional Ethical Committee.

A self-administered questionnaire consisting of 17 close-ended questions spread over 2 sections was used. A well-structured, pre-tested, self-administered questionnaire was adapted from Victoroff et al’s survey (2004). The content validity of questionnaire used in the present study was done by the experts in this field. The demographic details of participants were also collected. First section comprised of 9 questions which dealt with knowledge and attitude among dental students towards tobacco cessation counselling. A 3-point Likert scale was used to assess the response of participants. The second section comprised of 8 questions regarding practice of tobacco cessation counselling followed by students. 

The data collected through the study was subjected to descriptive analysis using SPSS version 20. Chi-square test was used to compare the two groups and p-value of <0.05 was considered as statistically significant. 

## Results

Amongst the 286 participants, 93 were interns and 193 were undergraduates. Approximately two-third of participants was females in both groups (Table 1).

**Table 1:** Distribution of Study Participants Based on Gender and Mean Age

Group	Male (%)	Female (%)	Mean age in Years (Standard deviation)
Under graduates (UGs)	29	71	22.1 (1.1)
Graduates	33	67	23.3 (0.62)

**Table 2:** Knowledge and Attitude of Dental Students Towards Tobacco Cessation Counselling

S. no	Questions		Year of study	Agree	Neutral	Disagree	p-value
1	It is within the scope of dental practice to:	a. Ask patient if they use tobacco	UGs Graduates	98.9% 98%	1.1% 1%	- 1%	0.39
		b. Advice patient to stop using tobacco	UGs Graduates	94.1% 99%	4.8% 1%	1.1% -	0.13

		c. Discuss benefits of stopping	UGs Graduates	98.4% 99%	1.6% 1%	- -	0.99

2	It is the dental professional’s responsibility to educate patients about the risk of tobacco use:w	a. Related to overall health	UGs Graduates	97.8% 96%	2.2% 3%	- 1%	0.35
		b. Related to oral health	UGs Graduates	99.5% 99%	0.5% 1%	- -	0.99

3	Dentist can act as a role model for society in the tobacco cessation program.		UGs Graduates	91.4% 97%	8.1% 3%	0.5% -	0.18

4	The dentist’s tobacco related habits will affect the impact of tobacco cessation counselling on patient.		UGs Graduates	88.7% 97%	9.1% 2%	2.2% 1%	0.05

5	Protocol for tobacco cessation counselling should be added to the dental curriculum.		UGs Graduates	83.3% 97%	14% 3%	2.7% -	0.003

6	Tobacco cessation counselling provided by a dentist would help the patient in quitting tobacco.		UGs Graduates	84.4% 96%	14% 2%	1.6% 2%	0.005

7	Dentist’s time can be better spent doing things other than stopping tobacco.		UGs Graduates	25.3% 20%	35.5% 21%	39.2% 59%	0.005

8	Patients don’t listen to dental students when they discuss tobacco usage.		UGs Graduates	52.2% 46%	31.7% 26%	16.1% 28%	0.05
9	Tobacco cessation counselling is ineffective unless the patient has a related health problem.		UGs Graduates	43% 28%	16.7% 14%	40.3% 58%	0.01


*P<0.05 as statistically significant; **p<0.01 as highly significant

**Table 3:** Responses to Practice of Tobacco Cessation Counselling Followed by Study Participants

S.no	Question	Year of study	Yes	No	P value
1	Do you take tobacco usage history from all patients?	UGs Graduates	91.4% 91%	8.6% 9%	0.91

2	Are you aware of the various forms of tobacco?	UGs Graduates	81.7% 96%	18.3% 4%	0.001

3	Have you ever done tobacco cessation counselling for any patient?	UGs Graduates	61.3% 87%	38.7% 13%	<0.001

4	Are you aware of the “5A’s” and the“5R’s” protocol of tobacco cessation counselling?	UGs Graduates	50% 79%	50% 21%	<0.001

5	Have you ever used the “5A’s” and the “5R’s” protocol while counselling on tobacco cessation?	UGs Graduates	17.2% 11%	82.8% 89%	0.16

6	Is information on tobacco cessation such as posters and pamphlets displayed in your institution?	UGs Graduates	83.9% 88%	16.1% 12%	0.34

7	Do you have access to tobacco cessation research literature via CD-ROM, books, internet etc?	UGs Graduates	50.5% 54%	49.5% 46%	0.57

8	Do you consume tobacco in any form?	UGs Graduates	15.1% 12%	84.9% 88%	0.47


*P<0.05 as statistically significant; **p<0.01 as highly significant

Table 2 illustrates knowledge and attitude of dental students towards TCC. Almost all study participants agreed that TCC is under the scope of dental practice and also admitted that it is the responsibility of dental professionals to educate patients for the same. Majority of undergraduates and young graduates believe that a dentist can act as a role model for society in the Tobacco cessation counselling and 88.7% of undergraduates and 97% graduates responded that dentist’s tobacco related habits will affect the impact of TCC. A significantly higher number of graduates (97%), as compared to undergraduates (83.3%), agreed that a protocol for TCC should be added to dental curricula. 96% graduates and 84.4% undergraduates believed that tobacco cessation counselling provided by dentist would help the patient in quitting Tobacco. This difference was statistically significant (p<0.005). A significantly higher percentage of graduates (59%) disagree to the fact that “dentist’s time can be better spent doing things other than stopping tobacco” as compared to undergraduates (39.2%). A significantly higher number of undergraduates (43%), as opposed to graduates (28%), believed that Tobacco cessation counselling is ineffective unless the patient has a related problem (p=0.01).

Table 3 demonstrates the response regarding practice of TCC where majority of study population took history of tobacco usage from all the patients. A significantly higher number of graduates (96%) are aware of various forms of tobacco as compared to undergraduates (81.7%). Majority of graduates have done tobacco cessation counselling whereas less than two-third of undergraduates have done the same. This difference between the two groups was statistically significant (p<0.001). A significantly higher number of graduates (79%) are aware of the 5A’s and 5R’s protocol of TCC as compared to the undergraduates (50%). But less than one-fifth of the study population have actually applied this protocol while conducting TCC. Majority of the study population said that information on tobacco cessation is displayed in their institution. 15.1% of undergraduates and 12% of graduates stated that they consume some form of tobacco.

## Discussion

Use of any form of tobacco always has a harmful effect on oral health as well as the overall health of the people. It has been found that most tobacco users failed to quit, if they do it without seeking any treatment or professional help (Mohanty et al., 2013). It has been proved in many studies that tobacco cessation programs are of great help to the users who want to quit tobacco (Mohanty et al., 2013 and Salman et al., 2014).

Dentists play an important role in the inter-disciplinary health professional team to support TCC. So, to act as a role model, it is imperative for dental professionals to understand their role. Our study shows that 91.4% undergraduates and 97% graduates agreed that dentist can act as a role model for society in tobacco cessation program. Various studies, showed a similar positive attitude in the students and they were aware of their responsibility to help others to quit smoking, thus, acting as a role model for patients and the community (Rikard et al., 2003; Dable et al., 2014). In contrast to these results, a study reported negative attitude of Nigerian dentists and clinical dental students towards TCC. Their pessimistic attitude was believed to be due to lack of training (Uti and Sofola, 2011). Researchers have proposed the need for implementing and embedding essentials of tobacco cessation counselling in the dental curriculum as this will not only promote importance of being tobacco free in greater population but also discourage their own habits (Sinha et al., 2012; Fotedar et al., 2013; Dable et al., 2014; Anjum et al., 2014). Results of the study conducted by Binnal et al., (2012) presented that study population under the age of 24 years showed more positive attitude towards TCC as compared to the older study population. This supports the notion of younger individuals being more optimistic and enthusiastic about what they do. In the present study, 97% graduates and 83.3% undergraduates were in favour of addition of TCC protocol in the dental curriculum. This overall positive attitude among the study subjects will help in improving their knowledge and boost their confidence towards TCC.

As many studies have suggested that TCC when done by a dentist will have a greater impact on the patients, therefore, dentists should devote more time towards it which will help in increasing the Tobacco quit rate. In our study, when asked if dentist’s time can be better spent doing things other than TCC, 39.2% undergraduates and 59% graduates disagreed. In contrast to this, majority of the Nigerian students felt that their time can be utilized in doing other things, which shows paucity of oral health and human resources as an estimated ration of dentist to patient is 1:41000, thus leaving them with busy clinical hours (Orenuga and da Costa, 2006). 

Almost half of the young dental students and graduates agreed that the patients don’t listen to dental students while they provide TCC, this may be due to skill barriers for providing TCC or hostile behaviour of patients and lack of motivation to quit tobacco among them. 

Although majority of the study participants of the present study said that they recorded Tobacco usage history from all of their patients, and it was evident from the data that majority of graduates (87%) and even fewer number of undergraduates (61.3%) have done tobacco cessation counselling for their patients. This statistically significant difference in the data shows that as graduates are more experienced, better versed with various forms of tobacco, their side-effects, feel more confident to do TCC as opposed to the undergraduates. In a study on Greek students similar results were found, that they possess significant knowledge about the health effects of tobacco and consider that it is duty of dentist to advise patients about tobacco use (Polychonopoulou et al., 2004). 

A negative attitude and discouragement towards tobacco consumption has been seen in the present study among the undergraduates and graduates, this is impressive to know and could be due to various discussions and information regarding tobacco forms and its deleterious effects on health.

5 major steps (the “*5A’s*”) for intervention of tobacco usage is important part of primary care setting. It is of great importance for a dental professional to “*Ask*” the patients if they consume tobacco, “*Advice*” them to quit, “*Assess*” willingness to make an attempt to quit, “*Assist*” the patient to make an attempt to quit and “*Arrange*” for follow-up to prevent relapse (Warnakulasuriya, 2002, Monson and Engeswick, 2005; Salman et al., 2014). In our study, 87% graduates admitted that they have done tobacco cessation counselling but only 11% of them have practically applied the 5A’s and the 5R’s protocol. This shows that insufficient skills are a barrier for providing appropriate TCC (Ehizele et al., 2009). Therefore, more emphasis should be laid on conveying information regarding TCC along with the use of evidence based teaching (Yip et al., 2000) so that the future clinicians will become more confident and well versed with the protocol and thus, enhancing the long-term quit rates attributable to clinician’s efforts.

In conclusion, our study revealed that majority of the students and graduates are willing to provide tobacco cessation counselling to the patients and are also eager to learn and practice the same with the latest protocol and methods of TCC. However it is found that due to didactic instructions on oral health and lack of competence on the practical front to intervene tobacco in the dental curriculum, many of the study participants are not able to give the TCC according to the protocol, and thus feel deficient. The present study concludes that there is a need to change the dental curriculum and motivate the young graduates and students about TCC. This will develop a more professional competence and helps to encourage the dental professionals in developing a preventive mind set about tobacco use. Thus it will help in enhancing the long-term rates of quitting tobacco habits among patients, which will prove beneficial in controlling tobacco related diseases in near future.
